# Scalable Agile Framework for Execution in AI for Medical AI Ethics Policy Design in Small- and Medium-Sized Enterprises

**DOI:** 10.2196/80028

**Published:** 2026-02-25

**Authors:** Ion Nemteanu, Adir Mancebo Jr, Leslie Joe, Ryan Lopez, Patricia Lopez, Warren Woodrich Pettine

**Affiliations:** 1 Nemsee LLC, San Diego, CA, United States; 2 Data Science Alliance, San Diego, CA, United States; 3 Department of Psychiatry, University of Utah, 501 Chipeta Way, Salt Lake City, UT, 84108, United States, 1 801-583-2500; 4 Mountain Biometrics, Inc., Salt Lake City, UT, United States

**Keywords:** digital health, eHealth, artificial intelligence, machine learning, medical ethics, small business, agile methodology, software development life cycle, risk assessment, fairness metrics, transparency, trustworthy AI

## Abstract

Artificial intelligence (AI) is transforming patient care, but it also raises ethical questions, such as bias and transparency. While a range of well-established frameworks exist to guide responsible AI practice, most were designed for academic or regulatory settings and can be hard to operationalize within fast-moving, resource-limited small and medium-sized enterprises (SMEs). We report on the collaborative design of the SAFE-AI (Scalable Agile Framework for Execution in AI), an approach that embeds ethical safeguards, including fairness, transparency, responsibility metrics, and continuous monitoring, directly into standard Agile development cycles. In keeping with established Agile principles, SAFE-AI provides “just enough structure” to integrate ethical oversight into existing workflows without prescribing extensive new governance layers. Similar to other Agile frameworks, such as Scrum, which is described as a “lightweight framework” designed to help teams solve complex problems through iterative learning and minimal process overhead, SAFE-AI aims to remain practical for organizations that may not have dedicated ethics or compliance staff. Rather than simplifying technical methods, SAFE-AI simplifies when and how ethical review is triggered and documented, making responsible AI practices feasible even in environments with limited ethics, governance, or compliance resources. SAFE-AI assumes the presence of qualified data scientists and engineers, and it does not replace the need for statistical or technical expertise but instead provides a lightweight structure for coordinating and documenting work that those experts already perform. We followed a design-science, practice-oriented approach over 20 weeks. After a discovery workshop, a cross-functional team was assembled that included SME employees, ethics researchers, and academic partners. The SME’s role was limited to informing design constraints and feasibility considerations during the co-design phase. No operational pilot or production deployment was conducted as part of this study. To reduce the risk of internal design bias and improve generalizability, we also consulted external stakeholders through structured feedback sessions, including clinicians, health care domain experts, and regulatory specialists. Their feedback was incorporated into each prototype-feedback cycle, ensuring that priorities reflected not only the SME’s immediate context but also broader clinical and regulatory perspectives. The co-design process produced a 4-phase SAFE-AI life cycle: discovery, assessment, development, and monitoring. SAFE-AI’s phase-specific checklists meld acceptance, fairness, and transparency metrics into each Agile sprint. A novel scenario-based probability analogy mapping method was added to translate model risk and uncertainty into plain-language narratives for nontechnical stakeholders, forming the framework’s core “responsibility metrics” layer. SAFE-AI is presented as a proposed framework showing that meaningful ethical safeguards can be embedded easily within common workflows used by SMEs that already use basic Agile or iterative development practices. Its checklist-driven phases and automatic review triggers provide a defensible way to track fairness, transparency, and responsibility throughout the model lifecycle.

## Introduction

### Background

Artificial intelligence (AI) systems deployed in health care, whether directly in clinical decision-making or through supporting technologies, carry significant ethical responsibilities. Their outputs can influence diagnoses, treatment options, operational workflows, and ultimately patient well-being. For organizations developing or integrating these tools, including small and medium-sized enterprises (SMEs) that support patient care, ethical considerations, such as fairness, transparency, and accountability, are essential from the outset.

While significant academic work has explored the ethics of deploying AI in medical decision support systems, these insights are often inaccessible to SMEs [1-6]. The complexity of AI ethics, including concepts like fairness, transparency, and accountability, presents a significant barrier, as does the prohibitive cost of dedicated ethics teams. Furthermore, many existing toolkits lack the practical guidance or real-world case studies necessary for SMEs [7,8]. Additionally, embedding ethics into AI development can be transformative for SMEs. Investments in ethical governance contribute to the value of intangible assets, such as intellectual property, loss aversion, brand reputation, and organizational goodwill. In health care markets, where regulatory trust and patient safety are paramount, demonstrable ethical alignment can also serve as a differentiator that attracts ethically aware customers, clinical partners, and investors. As a result, operationalizing AI ethics is not merely a compliance strategy but a value-creating asset for emerging technology companies. Proactive approaches tend to emphasize ways in which investments drive important intangibles that are reflected in a differentiated reputation and robust corporate culture. Mature firms link these intangible outcomes directly to investments in AI ethics and governance, using criteria, such as recognition of industry leadership and support of environmental, social, and governance efforts, as well as the long-term ability to manage risks [9].

In April 2024, HHS OCR (Department of Health and Human Services - Office for Civil Rights) clarified that decision-support tools, including AI, must not produce discriminatory outcomes under §1557 of the Affordable Care Act [10]. Outside software as a medical device (SaMD) pathways, comprehensive federal requirements remain unsettled. The US Food and Drug Administration (FDA) oversight and emerging guidance coexist with evolving proposals [11-13]. In this environment, organizations must anticipate stricter expectations while operating with practical, defensible governance. SAFE-AI (Scalable Agile Framework for Execution in AI) offers a pragmatic path for SMEs to meet today’s expectations and prepare for tomorrow’s.

To facilitate SMEs in addressing this challenge, we introduce a simplified framework in alignment with established Agile principles for responsibly integrating AI in medical settings. SAFE-AI aims to provide “just enough structure” to guide ethical oversight without imposing extensive new governance requirements, consistent with industry definitions of lightweight frameworks, such as Scrum, which emphasizes minimal process overhead and iterative adaptation [14,15,16]. Using this framework will help ensure SMEs develop medical AI solutions that meet the standard of large hospital systems and prioritize patient care.

Emphasizing AI responsibility, SAFE-AI addresses fairness, transparency, and ethical deployment in a manner proportional to organizational maturity and available expertise. A well-defined process facilitates collaboration between health care practitioners and data scientists, ensuring that ethical considerations, fairness, and equity are systematically assessed and documented. This approach ensures that each phase of AI development aligns with these critical values, leading to more effective and responsible AI tools for health care.

### Study Design and Project Context

This work followed a design-science, practice-oriented methodology to develop SAFE-AI, a scalable internal governance framework intended for broad adoption by SMEs building medical-AI products. The project was a collaboration among Medical Timeseries Networks (MTN; an SME), the Data Science Alliance (DSA), Nemsee LLC, and the University of Utah. MTN served as the primary design partner, while DSA, Nemsee, and academic partners contributed ethics, regulatory, and methodological expertise. MTN’s role in this study was limited to articulating operational constraints, workflow realities, and feasibility considerations during framework development; no operational deployment or live pilot was conducted as part of this study.

The purpose of the consortium was to embed ethics, regulatory, and methodological expertise into the SAFE-AI framework itself, rather than to provide ongoing expert participation in day-to-day development workflows.

## Informal Evidence Gathering

Between December 2024 and April 2025, the design team conducted an ad hoc search of peer-reviewed literature, regulatory guidance (eg, FDA, SaMD, Good Machine-Learning Practice [17], HHS §1557 rule [15], and EU (European Union; AI Act drafts) [18], and industry white papers. Searches were performed opportunistically in PubMed, Scopus, IEEE Xplore, and gray-literature repositories as questions arose during development. All relevant sources were captured in a shared Zotero library and tagged by topic. Key findings were reviewed in weekly design committee meetings.

## Framework Outputs

The co-design process yielded:

1. A 4-phase life-cycle diagram (discovery, assessment, development, and monitoring)
2. Stage-specific checklists covering acceptance, fairness, and transparency metrics
3. Practical implementation guidance (eg, role assignments and artifact templates) for each phase.

## Consensus and Documentation

All design decisions were made through real-time discussion until unanimous verbal agreement was reached. No formal voting or Delphi procedures were used. Version history, meeting minutes, and rationale for changes were maintained in a locked Google Drive folder that serves as the project’s audit trail.

## Future Work: Planned Pilot Implementation and Evaluation

The following pilot implementation and evaluation activities are planned for future work and were not conducted as part of the present design study. SAFE-AI will be applied to 2 upcoming MTN initiatives, including a workflow-orchestration product and a data-engineering pipeline. An evaluation plan was prespecified to capture:

1. Process metrics: time (hours) required to complete each SAFE-AI phase.
2. Governance metrics: count and category of fairness metrics defined per project.
3. Usability: postimplementation survey of participating staff (5-item Likert scale; 1=strongly disagree, 5=strongly agree).

Descriptive statistics will summarize these data after the first pilot quarter.

## Ethical Considerations

The project involved policy development only; no human participants or identifiable health data were used. Institutional Review Board (IRB) oversight was therefore not required.

## SAFE-AI Ethical Evaluation Framework

**Figure 1.**
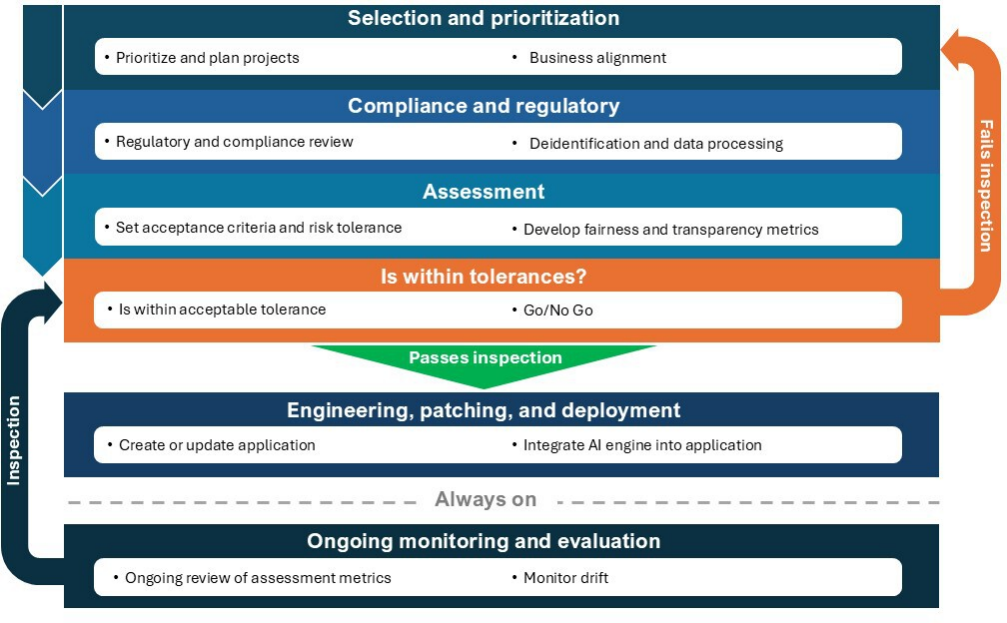
SAFE-AI (Scalable Agile Framework for Execution in AI) summary workflow, highlighting each core phase and feedback loop from prioritization to deployment and monitoring. AI: artificial intelligence.

**Figure 2.**
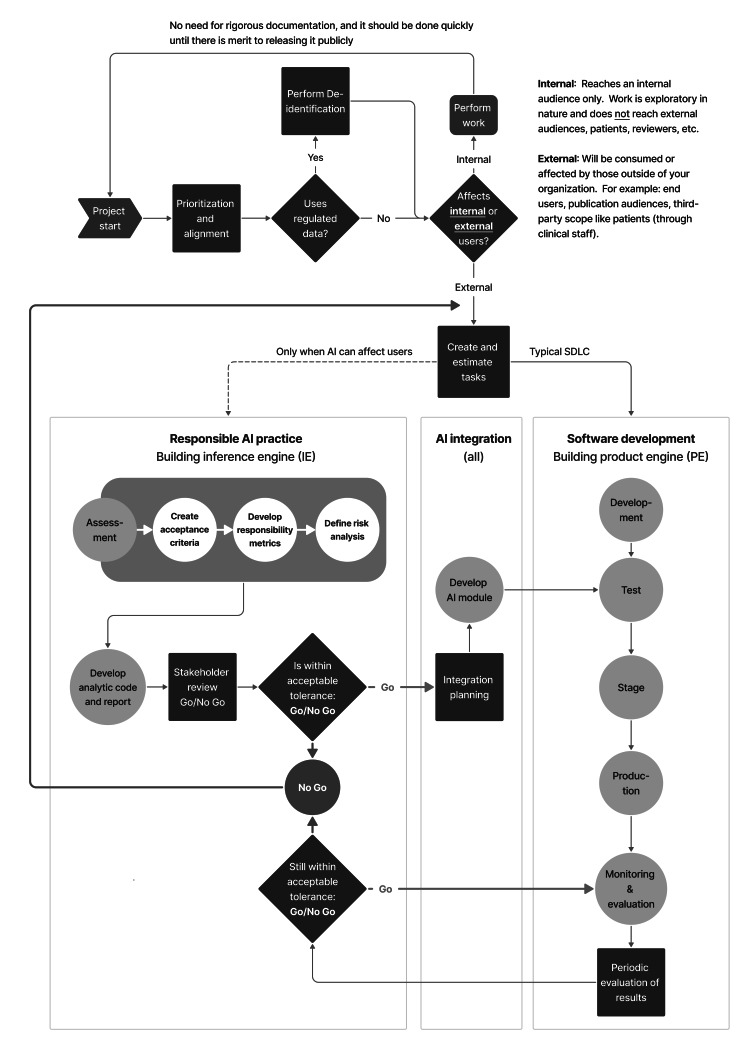
Detailed ethical evaluation process SAFE-AI (Scalable Agile Framework for Execution in AI) - conducted continuously throughout product development as illustrated above. The process begins with a discussion of priorities and alignment and proceeds through model building and inference implementation, emphasizing appropriate tolerance levels at each iteration. AI: artificial intelligence; SDLC: software development life cycle.

We present the SAFE-AI, which is a structured process designed to embed ethical evaluation into the technical development life cycle of AI products (summary in Figure 1, full process in Figure 2). A concise checklist distinguishing required and recommended steps is provided in Checklist 1 to support implementation consistency. SAFE-AI enters the workflow after business requirements or system objectives have been defined and prioritized, and tasks are ready for execution by the development team. It complements Agile and Scrum methodologies by introducing structured ethical oversight at critical stages without disrupting project momentum.

The SAFE-AI framework is not a one-time gate. It is designed to be re-entered at any point in the development lifecycle whenever changes occur that could materially affect the behavior, fairness, or transparency of the AI system. This includes retraining with new data, hyperparameter adjustments, updates to feature selection, or integrating external datasets.

Decisions to reactivate safe-ai should be made jointly by the product owner and technical lead, in consultation with the cross-functional team during natural Agile “sprint review” mechanisms when all the stakeholders are already present. This ensures that ethical checkpoints are triggered not only by technical changes (eg, retraining, parameter tuning) but also by product or stakeholder concerns (eg, shifts in clinical use cases). Embedding this responsibility into sprint reviews and release planning creates a consistent governance mechanism without requiring a separate oversight body.

Practitioners are encouraged to revisit SAFE-AI whenever there are any modifications to their models, regardless of perceived simplicity. Even seemingly minor technical tweaks may cause unintended shifts in model performance or equity. Further recommendations are outlined in the section titled “Quick Note on Model Updates and Considerations,” where we suggest re-evaluating the model against all the previously established metrics (refer to Part 2 [Assessment]) to ensure continued ethical integrity.

In this paper, the reference to a “data science team” is used as a generalized term to encompass data scientists and the other skilled roles and internal stakeholders that contribute to the project, including product and program managers, data engineers, analysts, statisticians, software engineers, and development operations personnel. However, when referring specifically to data scientists, we mean those roles that are directly responsible for researching and building the AI algorithms for use in the business and in their products.

### SAFE-AI: Part 1 (Discovery)

#### Determine if the Project Will Use Regulated Data

First, we will determine if the planned work will use or access regulated data. Organizations must comply with all relevant regional, state, federal, and local laws before any access and manipulation of data begins. Protected health information and personally identifiable information are among the many regulated data types. Existing acts, such as the General Data Protection Regulation (GDPR) and the California Consumer Privacy Act (CCPA) set specific compliance requirements.

Best practices include anonymizing or aggregating data to protect individual privacy. Additionally, informed consent is required for legally effective participation in research and must be obtained in accordance with the principle of respect for persons [19]. This principle ensures that individuals are given the opportunity to choose whether they wish to participate in research studies.

A comprehensive regulatory review should be conducted before any AI development begins. If specific handling techniques require documentation, they should be securely stored and referenced throughout development. When designing systems that impact external audiences, regulatory requirements should be listed as acceptance criteria and revisited at every development stage. If the same data are used repeatedly, the regulatory review can cover repeated use and should have a documented process (if necessary) to ensure compliance for future access and proper use. This will ensure that resources do not have to review the data every time it is accessed.

#### Determine if Scope of Work Impacts Internal or External Users

##### iDetermine External Effect

During typical prioritization meetings, it should be discussed whether the work will affect external stakeholders like patients, clinicians, or other health care providers. This is an important distinction since there are many tasks that should be done quickly and match the pace of the organization and do not require the extra burden of documentation. Realistically, not every task requires burdensome processes and documentation. This framework aims to only target the tasks that have a direct effect on the health care outcomes as a direct result of the product being developed. The first step in this process is a quick discussion during the planning meeting that determines the upcoming scope of work for the development team and clarifies if outputs can affect external stakeholders. Clarifications are detailed below.

##### Internal Stakeholders

Project work that includes exploratory research, feasibility studies, or internal management tools that do not directly impact patient outcomes can proceed without additional scrutiny. The process involves moving forward with the task as normal, without the need for additional scrutiny. Upon completion, the process is restarted from the beginning.

##### External Stakeholders

Work that affects those outside of your organization. For example, patients, clinicians, or other health care providers; end users, and publication audiences. In this case, the outcome of these tasks can or will have a direct impact on an external audience. Internal projects may also carry external implications*.* For instance, human resource initiatives that use protected employee data involve internal stakeholders, but mishandling that data can create risks that extend beyond the organization. The goal is to recognize when the project scope introduces potential external risk, even if it originates internally. In this case, the process involves moving on to the next step.

### Create and Estimate Tasks

This phase begins with breaking down high-level project goals into smaller, manageable user stories or tasks, as seen in Agile Software Development Processes. These tasks often span data ingestion, cleaning, feature engineering, model development, evaluation, and integration. During planning, the data science team estimates each task’s duration based on the scope of work for iterative delivery while leaving room for learning, model iteration, and integration into production workflows.

To ensure that ethical and social considerations are not deferred until the end of development, or left out entirely, this framework incorporates structured ethical reasoning into the task creation process from the start. The foundation for this integration draws from the RESOLVEDD process (Review, Estimate, Solutions, Outcomes, Likely impact, Values, Evaluate, Decide, and Defend), a structured ethical decision-making framework originally introduced by Pfeiffer and Forsberg [20,21]. RESOLVEDD guides practitioners through steps, such as reviewing the facts, estimating the ethical conflict, surveying alternatives, opening options to stakeholders, listing risks, valuing impacts, evaluating options, deciding, and defending the decision. While originally applied to workplace ethics, the logic of RESOLVEDD has broad applicability in responsible AI contexts.

Building on this foundation, Vakkuri et al. [22] introduced ECCOLA, a card-based toolkit tailored to Agile AI and autonomous systems development. ECCOLA does not follow an acronym, but its sprint-by-sprint format maps well to the RESOLVEDD process. It consists of a deck of 21 cards aligned to 8 core AI ethics themes (eg, transparency, accountability, and bias), each designed to provoke discussion, surface risks, and frame decisions during active development. The cards can be used in planning meetings to ensure that ethical issues are not only acknowledged but also actively scoped into the project as discrete tasks.

SAFE-AI adapts this card logic but moves one step further: rather than selecting from a predefined deck, teams create their own custom cards directly from the objectives and risks identified in the project. Each card captures the problem, the related ethical concern, and the concrete analysis tasks that will address it. These cards are then written into the backlog as user stories or research spikes, estimated during sprint planning, and assigned clear ownership. By embedding them directly into the backlog, ethical concerns are tracked with the same visibility and accountability as technical work. For example, a card generated from the objective “Minimize Gender Bias” might be translated into a user story with acceptance criteria tied to distributional checks, fairness metrics, and subgroup error analyses.

This approach converts abstract ethical principles into concrete, time-boxed actions that can be tracked and revisited throughout the sprint cycle. It ensures that responsibility is not just a post hoc evaluation, but a proactive part of scoping and estimating work. By embedding the card principle in this way, SAFE-AI enables collaborative assignment of each card and its tasks, supports ongoing discussion between stakeholders, and provides a shared method for everyone to follow. A simple template for these cards anchors the ethical problem with other “User Stories,” with the analysis tasks listed below (Table 1).

**Table 1.** SAFE-AI^c^ ethical card template for planning and backlog integration (eg, Minimize Gender Bias^a^^,^^b^).

What to do	Output
Training data balance. Quantify overall and conditional distributions by gender (and salient covariates)	Distribution tables and joint density plots
Split integrity. Verify gender balance across training, validation, and testing (or rolling production batches).	Per-split distribution summaries
Performance by subgroup: Report declared metrics by gender (eg, AUROC^d^, *F*_1_-score, PPV^e^, or NPV^f^ calibration) and compute gaps and confidence intervals.	Per-subgroup metric table Gap estimates Calibration plots
Downstream effect at acceptance testing: Compare consequential outcomes by gender (eg, referral or redirect rates) under final acceptance criteria.	Outcome distributions with statistical tests and effect sizes

a Do all genders have similar performance and minimized bias?

b Problem: The model may yield systematically different performance and error profiles across genders, a protected characteristic in health care decision support.

c SAFE-AI: Scalable Agile Framework for Execution in AI.

d AU: area under the receiver operating characteristic.

e PPV: positive predictive value.

f NPV: negative predictive value.

These tasks are customized per project and sprint. They should be time-boxed and assigned within the sprint backlog, ensuring fairness concerns are scoped into the same process as technical work.

### Integrating Into Agile Software Development Life Cycle

In practice, these SAFE-AI cards should be treated in the same way as user stories or research spikes within Agile planning. During sprint planning, teams can pull the relevant cards into the backlog and frame them as discrete work items with acceptance criteria, effort estimates, and clear ownership. This ensures that ethical questions are not abstract side discussions but concrete backlog items tracked alongside feature development. For example, a card highlighting fairness concerns may become a “bias audit story,” or a transparency card may generate a “model interpretability spike.” By embedding these cards directly into sprint discussions and backlog management, responsibility metrics are scoped, prioritized, and delivered with the same visibility as any other technical deliverable without introducing new governance layers or slowing iterative delivery.

**Figure 3.**
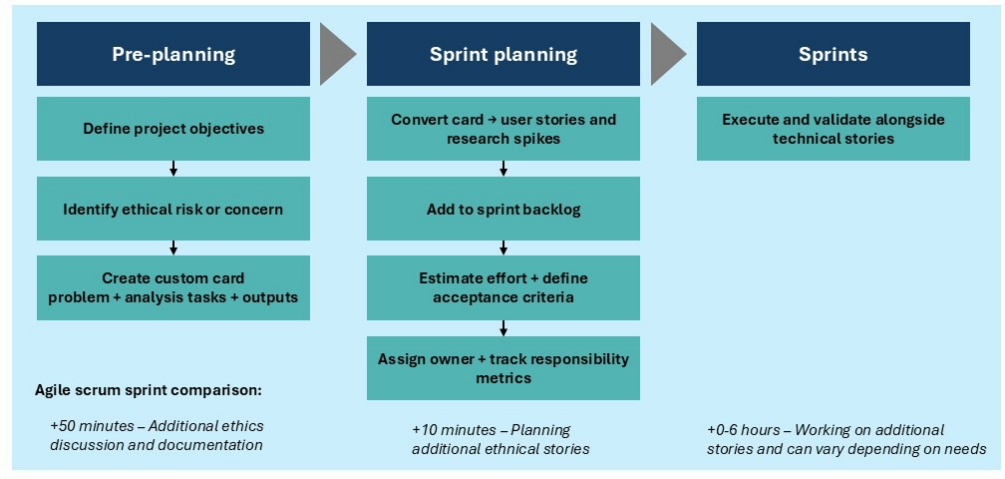
SAFE-AI (Scalable Agile Framework for Execution in AI) card workflow integrated into the Agile software development life cycle, including an estimate of the additional time required to incorporate SAFE-AI activities into standard Agile ceremonies. In preplanning, project objectives are defined, and ethical risks are identified, leading to the creation of custom cards that specify the problem, analysis tasks, and outputs. During sprint planning, these cards are converted into backlog items, such as user stories or research spikes, estimated, assigned, and given acceptance criteria. In the sprint phase, the ethical tasks are executed and validated alongside technical stories, ensuring responsibility metrics are tracked within the same Agile workflow with about 1-7 hours of additional time per sprint depending on the needs.

Importantly, integrating SAFE-AI into Agile requires only minimal additional ceremony. Teams typically spend about 50 minutes during preplanning to surface ethical risks and create the initial cards and approximately 10 minutes during sprint-planning ceremonies to refine and convert those cards into user stories or spikes (Figure 3). These brief additions occur within meetings that already exist, meaning the procedural overhead is minimal. The more substantial time investment (generally up to 6 hours within the sprint) is not overhead created by SAFE-AI itself but rather the time spent actually performing the ethical tasks, such as fairness checks, interpretability analyses, drift reviews, or documentation improvements. In many teams, these activities either occurred informally, inconsistently, or were never undertaken at all. SAFE-AI simply makes this work visible, trackable, and accountable by assigning it a discrete story with an owner and acceptance criteria.

SAFE-AI cards progress from the identification of ethical risks in preplanning to conversion into backlog items during sprint planning and finally to execution and validation alongside technical stories (Figure 3). This demonstrates that SAFE-AI adds only a small amount of process time while ensuring that ethical responsibilities are now explicitly planned, resourced, and delivered with the same rigor as technical features.

### SAFE-AI: Part 2 (Assessment)

#### Overview

This section occurs during the inference engine (IE) phase and outlines key steps the data science team, both technical and product stakeholders, should complete before progressing to development and integration. These steps ensure that AI IEs operate within defined boundaries of uncertainty and acceptable risk, aligning with ethical standards and strategic business goals.

It is important that the following steps be incorporated alongside traditional software testing as a routine part of development practice, with consistent attention to their execution. While these steps may initially appear to add time or complexity—requiring deeper evaluation, more test cycles, and added stakeholder input—when planned appropriately, these activities do not disrupt schedules. They are instead embedded naturally as part of the process and become part of the schedule.

The SAFE-AI framework is designed to be iterative and collaborative, ensuring that ethical assessments are embedded incrementally and transparently throughout the development lifecycle. Crucially, tasks are not imposed in isolation, as they are co-developed and agreed upon by all stakeholders, including data scientists, engineers, product leads, and domain experts, which keeps timelines aligned and priorities clear.

Ethical testing is treated with the same rigor and visibility as functional testing, making it easier to forecast and manage. While often overlooked due to perceived resource constraints or competitive pressure, these steps are essential to building systems that are robust, compliant, and trustworthy. The framework’s goal is to define the minimum necessary ethical safeguards required to ensure responsible AI while preserving agility and long-term strategic value.

There are 3 tasks that are especially relevant during the assessment phase, each of which is detailed below

#### Creating Acceptance Criteria

Acceptance criteria establish the conditions a system must meet before release. Borrowed from traditional software development life cycle (SDLC), they provide clear, testable expectations. In AI systems, this still begins with baseline functionality “Does the model work as intended?” For example, a clinical diagnostic tool might require ≥95% accuracy overall before being considered ready for integration into workflows. Such criteria help establish shared expectations and support regulatory alignment before AI models are integrated into clinical workflows. Atlassian describes acceptance criteria as “clear, concise, and testable statements that focus on providing positive customer results” [23,24].

In AI systems, especially those used in high-stakes domains like health care, defining acceptance criteria is not just a technical step—it is a normative one. The choice of what counts as “acceptable” performance can have real-world implications for safety, equity, and trust. It is therefore essential that these baseline criteria be co-developed with key stakeholders, including clinicians, patients, domain experts, and end users. AI engineers may assume they understand what matters most to users, but this assumption often overlooks critical perspectives—particularly around risk tolerance, fairness, and real-world utility. Engaging stakeholders in defining success conditions helps ensure that the system reflects the values and priorities of those it is intended to serve.

Acceptance criteria are only the first step. Once minimum functionality is defined, additional layers of responsibility metrics are needed to answer two further questions: “Does it work fairly, and is it understandable to those making directions on behalf of the company and those it serves?” Fairness and transparency extend acceptance criteria into the ethical domain, ensuring that the system not only functions but also functions responsibly.

#### Develop Responsibility Metrics

##### The Importance of Responsibility Metrics

Once baseline acceptance criteria confirm that a model works, the next step is to evaluate how it will work in practice. Responsibility metrics extend beyond functionality to address whether the system is fair, trustworthy, and aligned with stakeholder values. These metrics help organizations detect bias, communicate uncertainty, and ensure that model outputs can be understood and acted upon responsibly. Together, they provide the ethical guardrails that acceptance criteria alone cannot supply.

**Fairness Metrics**

Bias and equitable performance across groups within AI systems will be defined and measured. As BSA (Business Software Alliance [25]) states, fairness is subjective and requires selecting the most appropriate metrics for the system being developed. These metrics should be documented and reviewed collaboratively by subject matter experts and data scientists [25]. In practice, fairness metrics are used to detect and mitigate disparate impacts, ensuring that one group or class (eg, based on race, gender, age, or geography) is not disproportionately harmed or excluded compared to others. This may involve evaluating performance across subgroups, measuring disparities in false positives or negatives, or assessing equal access to beneficial outcomes. The goal is not only to identify statistical imbalance but also to actively choose metrics that reflect the ethical priorities and risk tolerances of the system’s intended users and affected populations [26-30]

**Transparency Metrics**

**Figure 4.**
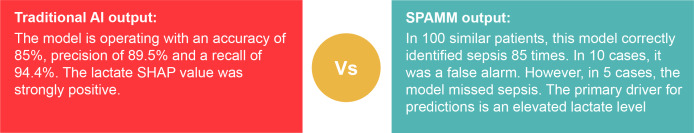
Example of scenario-based probability analogy mapping method compared to technical metrics. AI: artificial intelligence; SHAP: Shapley Additive Explanations; SPAMM: scenario-based probability analogy mapping method.

Metrics will be defined that focus on ensuring that AI model predictions are interpretable, explainable, and actionable by end users. One technique used in this framework is a scenario-based probability analogy mapping method (SPAMM)—a structured approach to communicating AI predictions by framing them as outcome narratives grounded in real-world context and relevant to the business goals. This method, which we refer to as the SPAMM, enhances transparency by mapping probabilities of outcomes related to fairness metrics and other acceptance and risk criteria to familiar analogies that illustrate the likelihood of different scenarios, the conditions under which errors occur, and their potential consequences. Rather than focusing solely on model internals, SPAMM presents predictions in narrative form - conveying not only what the model predicts, but why, how alternative outcomes compare, and which inputs most influence the result (Figure 4). This approach builds stakeholder trust by making uncertainty and risk both visible and relatable.

This approach is particularly important in high-stakes domains like health care, where decision-makers, such as clinicians, patients, or administrators must weigh the risks of false positives and false negatives. By highlighting the probability of misclassification and mapping those scenarios to familiar contexts, stakeholders can assess how often and under what circumstances the model may be wrong - and what the real-world consequences of those errors might be. For example, if a model is correct 85% of the time but consistently fails on a particular patient subgroup, this insight can guide mitigation strategies, prompt workflow adjustments, or justify additional human oversight. These dimensions of model behavior—error distribution, uncertainty, and impact—are often absent in standard AI evaluation pipelines.

Traditional interpretability tools like SHAP (Shapley Additive Explanations), LIME (Local Interpretable Model-Agnostic Explanations), partial dependence plots (PDP), and accumulated local effects (ALE) are valuable for understanding model behavior. SHAP, which is based on cooperative game theory, assigns each feature an importance value representing its contribution to a particular prediction—similar to calculating how much each player contributed to a team’s win. LIME works by perturbing the input data near a given instance and fitting a simple model to explain the local decision boundary. PDP and ALE methods help assess how the average model output changes when a single feature is varied, which supports global interpretability, especially when exploring relationships between inputs and outcomes.

These methods help data scientists and auditors understand what the model is doing, but they may not fully address the kinds of interpretive needs that arise when communicating overall impact with clinical or business stakeholders [31-35]. Technical performance metrics that evaluate true and positive rates, such as root mean square error, precision, and/or recall, can be difficult to translate to nontechnical audiences. As Ribeiro et al [36] noted, trust in machine learning (ML) models depends on the user’s ability to anticipate and understand model behavior in practical terms— not just accuracy, but reliability and risk.

As a result, we apply SPAMM, which adds a critical layer to the transparency toolkit by reframing predictive outputs in terms that mirror how people naturally reason under uncertainty. At its core, the method walks users through a model’s output by anchoring it to the specific input conditions that led to that prediction and by showing how often those conditions have historically produced similar outcomes.

Presenting outputs in this context helps stakeholders understand not just what the model predicted, but why it arrived at that decision, how likely alternative outcomes were, and how much confidence they can place in it. While this can become complex in models with high-dimensional input spaces or large numbers of interacting variables, the point is not to replicate the entire model logic, but to provide a real-world testing lens that frames predictions through the lens of practical “what-if” scenarios. This helps surface critical edge cases, increase interpretability, and build trust, especially in settings where human oversight and risk-based decisions are essential [37-39].

Crucially, this framing also enables teams to assign tangible costs to errors by explicitly asking “What can happen if this prediction is wrong?” and “What is the risk impact to the organization or patient?” For example, a false negative in a sepsis prediction model may delay critical treatment, leading to clinical harm, regulatory exposure, and reputational damage. A false positive in an insurance fraud detector may result in a wrongful denial of benefits, eroding user trust and increasing legal liability. By mapping these risks to predicted outcomes and associated variables, organizations can better understand which errors matter most, estimate their financial or operational consequences, and prioritize safeguards accordingly. Integrating these cost-of-error considerations into the transparency process transforms interpretability from a purely technical exercise into a tool for risk mitigation and strategic alignment [40,41].

Ultimately, this approach supports a more robust, stakeholder-centered view of transparency. It enables users to understand reliability, anticipate error, and make informed decisions based on modeled outcomes—an essential capability for ethical and accountable AI deployment in health care and other sensitive domains (Table 2).

**Table 2.** Illustrative acceptance criteria and responsibility metrics across common project types, including enterprise automation, consumer-facing artificial intelligence, and clinical decision support.

Project type	Acceptance criteria	Responsibility metrics
Enterprise automation	API^a^ response time ≤200 ms Workflow accuracy ≥98% across standard inputs	Fairness: Ensure no subgroup of users is excluded from automation Transparency: Apply SPAMM^b^ to communicate error risks, automation actions, and results, and maintain audit logs
Consumer-facing AI	Recommendation relevance ≥35% via click-through, ≥85 satisfaction Subgroup accuracy within ±5%, ≥10% Interaction with the next action	Fairness: Balanced recommendation exposure across demographic groups Transparency: Apply SPAMM to explain recommendations, actions, and results, and expose key features
Clinical decision support	Diagnostic accuracy ≥95% overall Subgroup disparity ≤5% across age, race, and gender	Fairness: Subgroup error analysis, including false positives and negatives Transparency: Apply SPAMM to frame predictions and risks in a clinical context

a API (application programming interface):

b SPAMM (scenario-based probability analogy mapping method):

This framework invites further development in areas such as visualization, user-centered metrics, and storytelling techniques for AI transparency. While this paper does not formalize the full methodology, it highlights the importance of designing explanation strategies that reflect how real-world users evaluate uncertainty, reliability, and relevance—not just technical performance.

In addition to defining fairness and transparency requirements, teams should specify the statistical methods that will be used later in monitoring to verify whether these metrics hold in production. For example, subgroup outcome disparities can be tracked using chi-square or Fisher exact tests, distribution shifts can be detected with Kolmogorov–Smirnov or Jensen–Shannon divergence, and overall population stability can be assessed with Population Stability Index (PSI). By embedding these statistical guardrails directly into the acceptance criteria, SAFE-AI ensures that monitoring processes in Part 4 are both preplanned and directly linked to the responsibility metrics established during development.

### Define Risk Analysis

Risk analysis should be conducted collaboratively, including consultation with relevant stakeholders like product teams, data scientists, and legal experts. There are many possible approaches, and the following are a few examples of structured risk analysis processes that already exist in the field of AI and health care.

1. The NIST (National Institute of Standards and Technology) AI Risk Management Framework provides structured guidelines for evaluating risks in AI systems, emphasizing trustworthiness and resilience through iterative assessments [42,43].
2. McKinsey’s “Derisking AI by Design” approach highlights the necessity of integrating risk management throughout the AI lifecycle, focusing on proactive monitoring and governance [44].
3. Stanford’s AI health care risk assessment framework outlines liability risks in medical AI applications, suggesting adaptive monitoring strategies to address unforeseen ethical and operational challenges [45].
4. The US Food and Drug Administration’s (FDA) Total Product Life Cycle (TPLC) framework ensures continuous oversight from AI model development through postmarket surveillance. The FDA writes how generative artificial intelligence (GenAI) presents potential risks that may require varying levels of risk controls for different applications, as is true of other technologies. For example, hallucinations, particularly those that may appear to be authentic outputs to users, may present a significant challenge in certain health care applications where highly accurate, truthful information is critical. They state how current evaluation approaches, such as those used to evaluate computer-assisted triage, detection, and diagnostic devices, may still be applicable for GenAI-enabled devices, albeit with additional supporting evidence. However, it may be challenging to determine the evidence that may be needed for certain GenAI implementations [46,47].

All of the above approaches include hazard identification, risk assessment, and mitigation strategies tailored to AI’s dynamic nature in health care. By incorporating structured risk evaluation, teams can proactively address potential harms and regulatory challenges. Key considerations include:

1. Misuse potential: Could the product be used incorrectly, leading to harm?
2. Error costs: What are the consequences of false predictions?
3. Societal implications: Are there broader ethical concerns?

Legal experts should be engaged to ensure compliance with evolving regulatory landscapes and provide specific tests and metrics that can be used to create risk boundaries for AI models that can help pass regulatory reviews as well as build trust with the public.

### Quick Note on Model Updates and Considerations

Once an IE has been approved and integrated into a production environment, it is common practice to revisit the model for improvements through techniques, such as retraining with new data, hyperparameter tuning, or feature modifications. While these changes may be perceived as incremental or routine, they carry the potential to significantly alter the algorithm’s behavior, calibration, and fairness properties. Even minor updates can reintroduce latent biases, shift subgroup performance, or affect the interpretability and reliability of outputs.

From an ethical standpoint, any modification to the IE, including changes to a single model or updates to the system of models, should be treated as a new model iteration when following the SAFE-AI framework, regardless of whether the foundational architecture remains unchanged. Although it is not necessary to recreate the entire ethical evaluation pipeline, it is considered best practice to reapply the original assessment metrics established during initial development (outlined in Part 2 [Assessment]). This allows teams to validate that the updated model continues to meet ethical expectations for fairness, transparency, and performance consistency.

In high-stakes domains, such as health care, the tension between deployment speed and responsible oversight is a persistent challenge. Rapid iteration is often essential for competitiveness; however, this urgency must be weighed against the risk of unintended harm. Ethical oversight should not be perceived as a barrier but rather as a safeguard that ensures continuity of ethical alignment across the model’s lifecycle.

Therefore, organizations are strongly encouraged to adopt a policy of proactive reassessment following any material change to an AI system. By incorporating these evaluations into their standard operating procedures, teams can preserve ethical integrity, mitigate risks, and maintain regulatory and stakeholder confidence.

### SAFE-AI: Part 3 (Development and Integration)

### Transition to Development

After defining metrics and performing the necessary reviews, the next step is to move from validated concepts into functional systems. This phase encompasses 3 key stages already defined in Part 2: the IE, the product engine (PE), and AI integration.

1. IE: As described earlier, the IE represents the culmination of algorithm development, transforming exploratory research into validated, production-ready code. Activities include hypothesis testing, feature engineering, model training, and validation, until the algorithm meets defined SAFE-AI thresholds for accuracy, risk, and subgroup reliability.
2. PE: In parallel, product engineers build the functional application layer. Here, validated models are embedded into the product architecture so they can import data, process it through the IE, and output results for downstream systems and users. Inputs, outputs, and thresholds are explicitly defined and jointly agreed upon. This work ensures the product is both technically robust and usable for stakeholders.
3. AI integration: Integration is the convergence point where the IE and PE are combined into a single, deployable system. At this stage, focus shifts from feasibility to operational viability, ensuring compatibility with data pipelines, embedding monitoring systems, and preparing the organization for deployment (training, regulatory compliance, user support, and related functions).

Together, these stages operationalize the SAFE-AI framework by moving from validated research into accountable, production-ready systems that can be deployed and monitored in real-world settings.

### Develop Analytic Code and Report

As part of the IE phase, data scientists transform exploratory research into validated inference code. This includes feature engineering, data exploration, model training and tuning, and refining the algorithmic process until it meets the established acceptance criteria and responsibility metrics. The key output of this phase is a report, which serves as the deliverable for review in the assessment and go or no-go decision.

This report functions as a consensus-building document: it (1) demonstrates how the model performs against the minimum functional standards defined in the acceptance criteria; (2) presents the results of fairness and transparency evaluations under the responsibility metrics, including subgroup performance, parity checks, error distributions, and interpretability narratives, such as SPAMM; (3) assesses overall risks and costs with implications for both users and the organization; (4) establishes a baseline of monitoring metrics and visualizations for long-term oversight; and (5) ultimately facilitates an informed go or no-go decision among stakeholders.

### Stakeholder Review Go or No-Go

After completing the above, the decision-making stakeholders should come together as part of an existing review or showcase discussion to review the report, analyze the results, and come to a consensus on whether or not the work is “good enough” to proceed to deployment. If the group determines that further refinement is needed, then the team updates the relevant metrics, documents the discussion, and begins another iteration of the assessment process within the same Agile cycle. Importantly, this review is not a new governance requirement being added to Agile. Most teams already make informal go or no-go decisions in sprint reviews, demos, or ad hoc leadership discussions. SAFE-AI simply formalizes and documents these joint decisions, so the rationale is transparent and traceable. It is important to include organizational leadership with the authority to make the executive decision in this regard, including people with ownership of balancing the risks of the algorithm against the needs of the organization.

AI developers and AI deployers should maintain a governance framework that is backed by sufficient executive oversight. In addition to developing and approving the substance of the governance framework’s policies, senior management should play an active role in overseeing the company’s AI product development lifecycle. For high-risk systems that may negatively impact people in consequential ways, company leadership should be accountable for making “go or no-go” decisions [25]. SAFE-AI does not add a separate approval gate, but rather aligns the accountability expected in high-risk AI with the existing cadence of Agile decision points, making explicit what is often handled implicitly.

### Integration Planning

Now that the inferential process has been completed and all stakeholders are aligned on the algorithm, the data scientist can work with the software engineers to lock down the deployment constraints. In some cases, this can be done before the “go or no-go” decision if inputs and outputs will not change significantly. It is important to have these completed before proceeding, although in practice, many teams already discuss these topics informally earlier in the development cycle. SAFE-AI does not introduce new tasks here, but simply formalizes conversations that often happen ad hoc, providing clearer documentation and coordination. It is good practice to perform integration planning as early as possible. However, performing this too early can create conflicts when the inputs and outputs change during the research phases. It’s best to perform this planning phase when there is good certainty that the inputs and outputs are well defined and not likely to change. By treating these steps as structured, documented alignment points rather than informal hallway conversations, teams avoid misunderstandings and reduce rework without adding meaningful overhead. For example:

1. Input or output contract: The data engineering process will need to know what data to create to feed into the algorithm and how often, while also asking for the algorithm outputs in return. This contract can be version controlled to say, “The system will always give you this data in this format, and your IE will always give certain data in a certain format in return.” This makes updating the algorithm in the future easier, which will be discussed later.
2. Update process: This should be a plan for which version of the production will integrate the first model. It is typical that an algorithm’s code may change more often than the production version and should have a process to deploy updates to the algorithm quickly without waiting for a full product release.

### Develop an AI Module

Now it is time to integrate the models as a new feature within the home application’s development environment that is now aligned with a planned production release. At this point, the work follows typical SDLC processes where the algorithms are engineered into the overall product architecture and involves performing all the other typical processes involved with a new feature release, such as marketing, educating stakeholders, etc

### Integration

Once the code is ready, developers move it into production, following the standard SDLC. It is essential to recognize that ML models and the broader application often have different deployment schedules, requiring a well-orchestrated Continuous Integration and Continuous Delivery process as illustrated in Textbox 1.

Textbox 1. **Defining distinct task categories that are relevant to the SAFE-AI (Scalable Agile Framework for Execution in AI) process.**

Developing product engine (PE): Traditional software development where the product architecture is built. These tasks are deterministic and repeatable, such as constructing databases, application programming interfaces, or user interfaces. Outcomes are binary (eg, schema exists or not; field matches or does not), and successful runs always yield identical results. The PE provides the stable scaffolding into which artificial intelligence (AI) components can later be embedded.

Developing an inference engine (IE): Unlike deterministic product code, IEs rely on models that incorporate randomness, sampling, or continuous learning, meaning the same code can yield different outputs across runs. This makes validation more complex, where predictions must be evaluated across multiple runs, data splits, and subgroups to ensure they fall within acceptable thresholds rather than simple pass or fail testing. As such, running the same code twice can produce different results. IE development spans from exploratory research (hypotheses, tuning cycles, and modeling notebooks) to validated inference code ready for integration.

Organizations often manage two parallel codebases during this phase: (1) research code is maintained by data scientists and (2) validated prediction engine code is used within the product engine. Only when the team confirms that the engine meets uncertainty and risk criteria does it become eligible for integration.

AI Integration (AII): The convergence point where prediction engine code is embedded into the product codebase. AI transforms research into an operational component that accepts input, runs the algorithm, and returns predictions through the product interface.

By the time the code reaches this phase, the model has been fully validated and is no longer being evaluated for feasibility—just functionality. The code is versioned independently and integrated in coordination with product releases to ensure seamless deployment.

**Figure 5.**
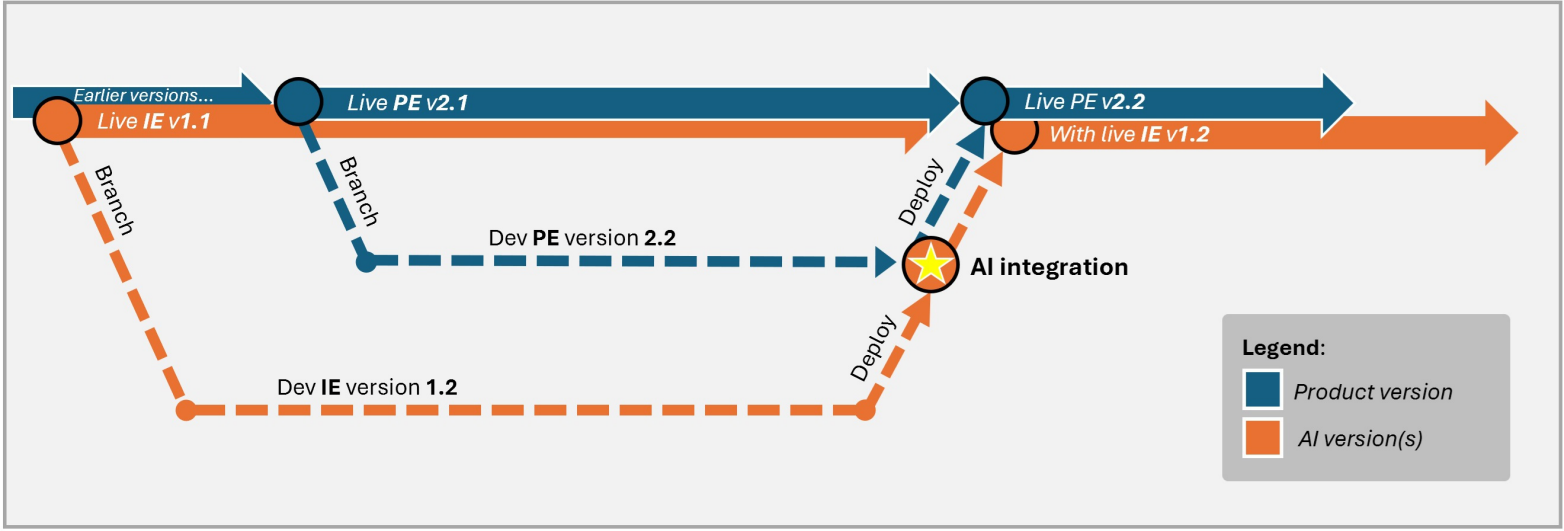
Example of product engine and inference engine version control with parallel development cycles. AI: artificial intelligence; PE: product engine.

To illustrate these differences, Figure 5 demonstrates how these phases operate in practice.

As shown, the IE and PE evolve on separate but parallel development tracks, each with its own branching and deployment cadence. For example, Live IE version 1.1 may remain in production while a newer Dev IE v1.2 is being validated, just as Dev PE v2.2 advances independently of earlier releases. The AII phase serves as the coordination point where validated AI models are embedded into product-ready systems, and inputs and outputs are aligned and locked. This moment (marked by the star icon in Figure 5) represents the culmination of both tracks, where a stable IE is paired with its corresponding product version for release. Once deployed, the system enters a new live state (eg, Live PE v2.2 with Live IE v1.2), ensuring that only approved, tested combinations reach end users. This disciplined integration workflow supports transparency, traceability, and accountability across the AI development lifecycle.

It is important to note that changes to the IE don’t always involve modifications to input or output formats. The structure of the data pipeline may remain consistent even as the underlying model evolves through algorithm updates, parameter tuning, or other enhancements. The purpose of the diagram is simply to illustrate that, at some point, the IE and PE must converge during the AI Integration phase. However, since IE updates can occur independently of PE deployments, it’s critical that any such changes, regardless of how minimal they may appear, still undergo the SAFE-AI evaluation process. This ensures that even behind-the-scenes model improvements are held to the same standards of validation, risk assessment, and accountability.

For example, ML models may need to be retrained and updated more frequently than full product releases. This raises important considerations, such as how to introduce new parameters or model updates without disrupting the existing environment.

Ensuring a smooth deployment process requires clear coordination between the data science and software development teams. These discussions should focus on versioning strategies, model refresh cycles, and seamless integration workflows to maintain system stability while enabling continuous improvements (Textbox 2).

Textbox 2. **Example illustration of SPAMM (Scenario-based Probability Analogy Mapping Method) in practice**

To illustrate SPAMM, the project is assumed to be a system that helps to triage emergency room visits for nonurgent patients only (not to include resuscitation or emergent patients) based on incoming patient data and demographics to try and reduce patient wait times and complications caused by long waits and overcrowding. The goal is to translate your inference engine’s preferred error metrics, such as recall, accuracy, root mean square error, and other technical metrics, into the real actions and events that will happen as a result of the predictions.

This is a demonstration example only. It illustrates how the SPAMM method can be applied, but it is not a real clinical analysis.

Step 1 (complete assessment steps): Before building scenarios, all relevant metrics and goals found in the acceptance criteria, responsibility metrics, and risk analysis steps in SAFE-AI (Scalable Agile Framework for Execution in AI) should be defined and documented. That will ensure the organization has a shared baseline of “what matters,” how to measure it, and the cost of getting it wrong.

Step 2 (translate acceptance criteria into scenarios): For a relevant metric, these should be associated with the simulated outcomes from the inference engine that is translated into how those predictions relate to the real-world actions and consequences of the model.

**Overall Scenarios**

- The Emergency Department receives about 152 “nonurgent” patients a day, with 4 out of 5 of those patients experiencing no change in how they would normally be treated.
- If all of the prescribed actions are implemented:

- About 15% (23/152) of these patients would end up waiting longer than they normally would, since the system would reprioritize other cases ahead of them.
- About 5% (8/152) of these patients would be classified as higher urgency than they normally would, meaning they would be escalated for faster treatment than under standard triage.
- Of the 15% (23/152) of patients who would experience longer wait times, only about 1 in 1000 would experience an adverse effect due to the delay (approximately 9 cases per year).
- For the remaining ~23 patients per day who waited longer but did not experience harm, their delayed care effectively freed capacity for more urgent patients. This reallocation reduced other ER-related adverse events by an estimated 5% (around 25 cases per year).

- **Demographic Scenarios** • Age: For every 100 older patients, about 8 are predicted incorrectly. These patients have a 50% higher chance of harm and accounted for nearly all of the adverse events caused by longer wait times. • Race: For every 100 Black patients, about 6 are predicted incorrectly, with about a 5% higher chance of harm. • Gender: There are no meaningful differences in error rates by gender.

**Conclusion**

- Most patients (121/152, 80%) are triaged the same way they would be under the current system.
- Roughly 8 patients per day are upgraded, meaning they receive care faster than before.
- About 24 patients per day are downgraded, which creates some additional risk, but only around 1 in 1000 of these cases leads to actual harm (about 9 cases per year).
- By comparison, the reallocation of resources reduces other ER-related harms by an estimated 25 cases per year, suggesting an overall safety benefit.
- The most significant concern is that the small number of adverse events disproportionately affects older patients, and Black patients show slightly higher error rates.
- These fairness issues, together with the rare but high-impact missed urgent cases, represent the biggest risks to the hospital’s reputation and liability. Smaller risks, such as false alarms or mild misclassifications, carry lower per-case costs but can still influence efficiency.

Overall Demonstration Conclusion: This example illustrates how a triage support system could reduce wait times and improve patient flow while also exposing the disproportionate costs of rare but severe errors and subgroup disparities. From a business perspective, the primary risks are tied to patient harm, liability exposure, and reputational impact. The exercise is not a real analysis but a demonstration of how SPAMM can be applied to create scenario-based narratives that connect acceptance criteria, responsibility metrics, and risk analysis into a single story for decision-makers.

### SAFE-AI: Part IV- Monitoring and Evaluation

System monitoring and evaluation at regular intervals can help ensure the prevention of potential harm creation or perpetuation. While initial model results may meet responsibility metrics during development, changes in data patterns or underlying system characteristics could cause deviations in production performance.

According to Data Science Alliance [48] and Guan et al [49], 5 important types of concerns exist when conducting monitoring:

1. Concept drift: Also known as model drift, this occurs when the patterns or relationships estimated by the model shift from their state at deployment. These shifts can manifest instantaneously, gradually, cyclically, or temporarily, potentially compromising the model’s effectiveness. This can occur abruptly (eg, new treatments altering outcome rates) or gradually (“AI aging”), requiring continuous recalibration [49].
2. Data drift: This phenomenon emerges when the distribution of production data significantly differs from the training data. While the negative impact of data drift on model performance typically develops slowly, its effects compound over time, leading to increasingly unreliable results [48].
3. Data integrity: Production environments may introduce unexpected variables, incompatible data types, or inconsistent measurements that differ from the model’s training environment. These integrity issues can severely impact the model’s accuracy and reliability, requiring immediate attention and correction [48].
4. Bias drift: Even a model developed with careful consideration for fairness and bias mitigation can become unfair in production due to bias drift. As societies evolve, existing biases may diminish while new ones emerge, requiring continuous monitoring and adjustment to maintain ethical performance [48].
5. Calibration drift: Highlighted by Guan et al [49] as a distinct concern in health care: a model may maintain discrimination but lose calibration, producing systematically over- or underestimated risk scores that undermine trust and safety [49].

The categories of drift outlined by Data Science Alliance [48] can be directly linked to statistical methods described by Guan et al [49], which provide concrete means of distinguishing meaningful changes from random variation. By pairing each drift type with corresponding identification strategies, it becomes possible to operationalize conceptual risks into measurable safeguards. Table 3 shows how SAFE-AI connects Data Science Alliance’s [48] framework of monitoring concerns with Guan et al [49] recommended statistical approaches.

**Table 3.** Statistical tests and drift identification strategies (adapted from Guan et al [49]).

Drift type and formula or test	When to use
Concept drift	
Cumulative Sum and Shewhart or SPC^a^ control charts	For monitoring performance metrics, such as AUC^b^ or calibration slope, over time to detect gradual “AI^c^ aging” or abrupt performance changes that are effective for real-time detection of sustained deviations.
Data drift	
Maximum mean discrepancy	For high-dimensional feature spaces (eg, latent embeddings from imaging or EHR^d^ data) It detects subtle covariate shifts.
Wasserstein Distance (Earth Mover’s Distance)	For comparing distributions of latent features, like in medical imaging or pathology data It is sensitive to spatial domain shifts
Kolmogorov–Smirnov test	For univariate continuous variables (eg, lab values and vital signs) It outputs a test statistic and a *P* value to assess whether the distributions differ significantly.
Jensen–Shannon divergence	For comparing feature or prediction distributions across temporal windows, be robust to temporal shifts.
Bias drift	
Chi-square test	For categorical variables (eg, sex, race, and hospital unit) Applied in metadata to detect subgroup distribution shifts
Fisher exact test	For small sample sizes where chi-square assumptions fail (eg, minority subgroup outcome analysis).
Calibration drift	
Calibration slope or intercept and dynamic calibration curves	For tracking whether predicted probabilities remain aligned with observed outcome frequencies.
Data integrity	
Rule-based validation and schema checks	For detecting anomalies, such as new, unexpected, or missing variable encodings and inconsistent measurement units

a SPC: statistical process control.

b AUC: area under the curve.

c AI: artificial intelligence.

d EHR: electronic health record.

To distinguish these forms of drift from random variation, Guan et al [49] emphasize the importance of embedding formal statistical testing within monitoring pipelines. Data drift can be identified using statistical distance measures, such as Maximum mean discrepancy or Wasserstein distance for high-dimensional features, Kolmogorov-Smirnov tests for continuous variables, and Jensen-Shannon divergence for monitoring temporal distributional shifts. Subgroup outcome disparities should be routinely assessed with categorical association tests, such as chi-square or Fisher exact test, ensuring fairness is maintained across demographic groups. Calibration drift requires continuous evaluation of calibration slope and intercept, or dynamic calibration curves, to confirm that predicted probabilities remain aligned with observed outcomes. For concept drift and temporal performance degradation, including the gradual loss of discrimination, sequential statistical process control methods, such as cumulative summation and Shewhart control charts, are effective for detecting “AI aging” in deployed systems (Table 3). In this way, the SAFE-AI framework operationalizes Guan et al [49] recommendations by mapping conceptual drift categories to concrete identification strategies relevant for health care contexts.

Operationalizing these methods requires automated pipelines that compute drift, calibration, and fairness metrics at scheduled intervals, with alerts triggered when thresholds are exceeded. However, detection alone is insufficient [49,50]. The need for multidisciplinary review is stressed, where data scientists, clinicians, and ethics experts interpret signals and determine whether retraining, recalibration, or workflow changes are warranted. Equally important is the structured documentation of drift events, calibration measures, and corrective actions, which provides transparency, supports regulatory alignment, and builds trust. Embedding such statistical guardrails within governance processes reduces false alarms and enables timely interventions before performance degradation compromises patient care.

Below are the recommended steps to implement an effective monitoring structure:

1. Automate monitoring and alerting: Deploy automated pipelines that calculate drift and fairness metrics at scheduled intervals. Integrate with alert systems (eg, dashboards, email, and Slack notifications) to flag deviations as soon as they occur. For example, trigger an alert if PSI >0.25 or subgroup accuracy difference >10%.
2. Scheduled reviews: Implement periodic review sessions (eg, monthly or quarterly) involving stakeholders (data scientists, clinicians, and ethics experts) to analyze monitoring reports and determine if intervention, such as model retraining or recalibration, is needed.
3. Document changes: Maintain a structured log (eg, an SQL table or Pandas DataFrame) capturing timestamps, versions, drift statistics, fairness scores, and actions taken.

Table 4 illustrates how SAFE-AI’s automated monitoring and documentation process operates in practice. The table represents a simplified monthly log of model performance and fairness metrics tracked through an automated pipeline. In this example, Model 1.2.1 initially performs within acceptable bounds, showing low data drift (PSI <0.10) and stable fairness scores.

**Table 4.** Example of monitoring statistics table.

Date	Model_ID	Data_Drift (PSI^a^)	Accuracy	*F*_1_-score	Fairness_Parity	Bias_Flag	Notes
January 3, 2025	1.2.1	0.07	0.86	0.91	0.94	Pass	Stable
January 4, 2025	1.2.1	0.08	0.81	0.92	0.93	Pass	Stable
January 5, 2025	1.2.1	0.11	0.79	0.89	0.90	Warning	Stable
January 6, 2025	1.2.1	0.15	0.74	0.85	0.88	Alert	Notified developer
January 7, 2025	1.2.1	0.19	0.68	0.80	0.84	Fail	Failed
January 8, 2025	1.3.0	0.05	0.88	0.92	0.95	Pass	Updated

a PSI (population stability index):

By May 2025, drift begins to rise and accuracy drops slightly, triggering a warning flag. In June, the system automatically issues an alert notification to developers as drift and subgroup disparity increase. By July, both performance and fairness fall below defined SAFE-AI thresholds, producing a fail status that prompts model retraining.

A month later, version 1.3.0 was deployed. The updated model restores accuracy, stability, and fairness parity to acceptable levels, confirming successful remediation.

This example demonstrates how SAFE-AI’s automated monitoring, alerting, and documentation pipeline detects early signs of degradation, escalates issues for review, and records corrective actions, creating a transparent, auditable trail of model health over time.

1. User feedback integration: Establish channels for end-user (clinician and patient) feedback regarding both model performance and explainability. Use structured surveys and focus groups to gather qualitative data that can inform further improvements.
2. Iterative improvement: Based on audit findings and feedback, implement retraining or fine-tuning cycles that reincorporate recent data. Validate updated models against the established responsibility metrics before redeployment.

Effective system monitoring and evaluation represent crucial components of responsible AI deployment. Potential issues can be identified and addressed early on with the right tools in place. This proactive approach, combined with structured feedback mechanisms and continuous improvement processes, helps ensure that AI systems remain both technically sound and ethically aligned with their intended purposes.

Much of the monitoring and evaluation effort falls within the “0‐6 hours” of additional sprint time associated with SAFE-AI integration (Figure 3). Importantly, this represents the structured version of work that most teams already perform informally, such as checking whether performance has shifted, manually reviewing error cases, or responding to unexpected model behavior in production, rather than a new category of tasks introduced by SAFE-AI. This framework formalizes existing monitoring responsibilities, provides statistical methods to replace ad hoc practices, and ensures that time spent diagnosing drift, fairness issues, or degraded calibration is tracked as intentional sprint work with owners and acceptance criteria. In this way, the monitoring step reflects not additional governance layers, but a clearer, more accountable articulation of work that must occur to sustain responsible AI performance over time.

## Discussion

### Balancing Ethical Oversight With Business Objectives

The SAFE-AI framework distinguishes itself by aligning ethical rigor with business priorities and development speed. Whereas existing frameworks, such as ECCOLA [51] or academic or regulatory models, are often perceived as prescriptive or cumbersome, SAFE-AI integrates oversight into Agile workflows without slowing delivery. Ethical review is framed not as external compliance but as a strategic safeguard against regulatory, reputational, and financial risks.

SAFE-AI frames ethical assessment not as an overhead cost, but as a strategic risk mitigation process that helps ensure model integrity, maintain user trust, and prevent potential regulatory, reputational, or financial harm. SAFE-AI is purposefully designed to complement the natural rhythms of modern software and AI product development by integrating ethical review seamlessly into existing Agile and Scrum workflows.

One of the primary distinctions of SAFE-AI is its emphasis on efficiency without compromising ethical standards. Rather than exhaustive checklists or governance layers, SAFE-AI establishes the minimum necessary safeguards to preserve responsibility while remaining agile. This proportional approach makes it especially practical for SMEs with limited resources.

A key aspect of this balance is recognizing that the time SAFE-AI introduces into the development cycle, such as the additional 6 hours of work during a sprint, is not a totally new burden but a structured articulation of tasks that development teams typically perform informally. Most teams already spend time investigating unexpected model behavior, resolving data inconsistencies, responding to stakeholder concerns, or informally reviewing fairness or performance anomalies. SAFE-AI does not create totally new categories of work, but it simply moves these ad hoc activities into the sprint backlog, where they can be estimated, tracked, owned, and completed with the same discipline as technical tasks to ensure they are completed. By making previously invisible labor explicit rather than adding new governance layers, SAFE-AI preserves development speed while ensuring that ethical safeguards are delivered consistently and transparently.

A central innovation is the SPAMM. Unlike SHAP, LIME, or other interpretability tools that emphasize technical explanations, SPAMM translates model behavior into real-world narratives and cost-based analogies. This enables nontechnical stakeholders to grasp the implications of model errors, facilitating informed trade-offs between ethical risk and business priorities.

SAFE-AI also emphasizes iterative oversight. AI systems evolve through retraining, tuning, and updates. SAFE-AI requires previously defined acceptance and responsibility metrics to be revisited after each substantive change. This ensures updates do not reintroduce bias or compromise fairness while avoiding unnecessary rework.

Finally, SAFE-AI frames ethical responsibility as a shared duty across cross-functional teams, embedding accountability within everyday workflows rather than delegating it to specialized ethics committees.

### The Critical Role of Risk Interpretability and Metric Development

A core principle of ethical AI development in health care is ensuring that the inherent risks of automated decision-making are both interpretable and measurable. Modeling risk interpretability is not a secondary concern but a foundational necessity for responsible AI deployment. Without clear and agreed-upon mechanisms for understanding how an algorithm’s predictions translate into real-world outcomes, health care providers and affected individuals are left to navigate an opaque and potentially harmful system.

The introduction of the SAFE-AI framework addresses this challenge directly by developing probability analogy methods to bridge the gap between complex model behavior and the practical, human-driven ethical decision-making processes within health care settings. Unlike traditional explainability tools, which often focus exclusively on mathematical metrics or technical explanations, while emphasizing narrative-driven, probabilistic scenarios that map model behavior to understandable analogies. This approach acknowledges the reality that nontechnical stakeholders, including clinicians, patients, and regulatory bodies, must ultimately be able to evaluate and engage with the outputs of AI systems in order to trust them.

The significance of this method is especially relevant during the assessment phase of SAFE-AI. Setting responsibility metrics is not merely a technical exercise but an ethical obligation. Without these metrics, it is impossible to contextualize and evaluate how well the algorithm is performing against real-world risks. The development of responsibility metrics, including fairness and transparency metrics, must occur in tandem with risk interpretation strategies to ensure that metrics are not abstract or arbitrary but rather aligned with the actual impact on the user population.

Additionally, one of the most overlooked sources of ethical risk in AI systems is introduced not during the initial development but during subsequent algorithmic updates, such as model retraining or hyperparameter tuning. Even seemingly minor updates can alter the balance of an algorithm in subtle ways that inadvertently introduce ethical imbalances or exacerbate existing disparities. As described in earlier sections, SAFE-AI mandates that any update to an existing inference engine—whether through additional training data, tuning, or rebalancing—should be treated as a new project cycle if there is any risk that new training data can create predictions that fall out of bounds. Although previously established assessment metrics can and should be reused, they must be explicitly re-evaluated to verify that any modifications have not unintentionally undermined fairness, transparency, or safety.

This approach balances the need for iterative improvement with the ethical imperative to avoid harm. Too often, pressure to deploy quickly or incrementally improve system performance results in bypassing critical ethical assessments; however, many algorithms use random samples as part of their retraining process, and a quick iteration can inadvertently create vastly different outcomes. SAFE-AI stresses that, even in fast-paced environments, evaluating updated models against established metrics is essential. The cost of skipping this step is not merely technical debt but ethical liability, with real consequences for patient safety and equity.

Finally, it is important to emphasize that the development of these metrics and risk interpretability mechanisms cannot be left solely to technical teams. The most effective way to safeguard against ethical harm is through multidisciplinary collaboration. Risk interpretation is inherently contextual, requiring input from health care professionals, regulatory experts, ethicists, and product stakeholders who understand the social and operational realities in which the AI system will function. This collaborative approach ensures that metrics are not only technically valid but also ethically relevant and meaningful to the people most impacted by the model’s behavior.

By prioritizing transparency, risk interpretability, and ongoing evaluation using the approaches outlined in the “Develop Responsibility Metrics” section, the framework supports the development of AI systems that are not only effective but also fair, accountable, and ethically sound.

### Validating the Framework in Real-World Health Care AI Settings

To assess SAFE-AI’s real-world relevance and impact, future work should focus on comprehensive field-testing across diverse health care AI applications. The empirical analysis would involve implementing the SAFE-AI framework with SMEs developing various medical AI systems, including diagnostic support tools, patient outcome assessment, and wound trajectory prediction. Using a pre-post intervention design, these studies would evaluate the framework’s impact on development practices, fairness outcomes, and organizational dynamics in real-world health care AI deployments.

Within health care settings, the field studies would specifically examine how the SAFE-AI framework performs across various clinical applications, where ethical considerations regarding patient care and outcomes are particularly critical. The research would investigate how effectively SAFE-AI addresses issues, such as training on biased datasets, transparency requirements, preventing provider overreliance on AI systems across different clinical contexts, and how to ensure its seamless integration into existing processes. By studying implementation across diverse medical specialties, it is possible to assess SAFE-AI’s adaptability to varying clinical workflows, data types, and ethical priorities.

The proposed implementation process would include a baseline assessment to capture preframework metrics, structured implementation of the responsibility protocols, and postimplementation evaluation at multiple intervals. This systematic approach would allow for the identification of potential adoption challenges, including resource constraints, workflow disruptions, and organizational resistance. Field testing would evaluate various implementation strategies, such as gradual integration, iterative refinement, and leadership engagement models, to determine which approaches most effectively support framework adoption while maintaining rigorous ethical standards.

Building on these future validation efforts, the framework presented here offers a practical approach to embedding ethical considerations throughout health care AI development while maintaining efficient workflows. By transforming abstract principles into concrete processes with measurable outcomes, this framework helps bridge the gap between technical implementation and ethical governance. As health care AI systems become increasingly integrated into clinical decision-making, such structured approaches to responsible development will be essential for building trustworthy systems that genuinely advance patient care without introducing new forms of bias or harm.

### Comparison With Existing Frameworks

Table 5 situates SAFE-AI alongside ECCOLA and CARE-AI (Collaborative Assessment for Responsible and Ethical AI Implementation), two of the most prominent ethical AI frameworks. While ECCOLA provides broad governance prompts and CARE-AI emphasizes health care assurance cases, both approaches are resource-intensive and often external to fast-paced product development. In contrast, SAFE-AI was co-designed with practitioners to embed ethical safeguards directly into Agile or Scrum sprints, collaboratively customize responsibility metrics to the project at hand, and lower adoption barriers for SMEs. While the development of SAFE-AI benefited from an expert consortium, this involvement intentionally front-loaded ethical and regulatory complexity into the framework design so that downstream SME users can apply its artifacts without requiring comparable ethics or academic partnerships.

**Table 5.** Example of SPAMM^a^ compared to technical metrics.

Criteria	ECCOLA	CARE-AI^b^	SAFE-AI^c^
Origin or context	Academic or consortium-led, broad AI^d^ governance guidelines	Health care–oriented ethical assurance	SME^e^-practical, co-designed with practitioners
Workflow integration	Provides ethical prompts or checklists, but external to development sprints	Process guidance tied to assurance cases; heavy documentation	Fully embedded into Agile or Scrum sprints with sprint-based checkpoints
Fairness coverage	High-level bias prompts, no quantitative thresholds	Addresses equity in health care safety cases	Defines fairness metrics with subgroup thresholds and SPAMM narratives
Transparency coverage	Suggests explainability but lacks concrete methods	Assurance-case documentation for regulators	Combines methods like SHAP^f^ or LIME^g^ with SPAMM for all domains (scenario-based narrative analogies)
Monitoring	Minimal; assumes postdeployment audits	Focus on compliance reporting and auditability	Continuous monitoring with statistical drift detection triggers
Adoption burden	High (lengthy prompts or checklists and steep learning curve)	High (documentation intensive; requires assurance specialists)	Low to moderate (checklists and metrics integrated into existing development tools)
Scalability to SMEs	Limited; designed for larger institutions	Limited; high compliance load	Explicitly designed for SMEs; pragmatic, “minimum necessary” safeguards

a SPAMM: scenario-based probability analogy mapping method.

b CARE-AI: (Collaborative Assessment for Responsible and Ethical AI Implementation).

c SAFE-AI: Scalable Agile Framework for Execution in AI.

d AI: artificial intelligence.

e SME: small and medium-sized enterprise.

f SHAP: Shapley Additive Explanations.

g LIME: Local Interpretable Model-Agnostic Explanations.

A key differentiator is SAFE-AI’s explicit use of fairness metrics and SPAMM narratives to operationalize equity and transparency across domains, whereas ECCOLA and CARE-AI stop at higher-level guidance. Similarly, SAFE-AI integrates continuous monitoring with statistical drift detection, allowing ongoing oversight without the compliance burden of full assurance-case documentation.

Taken together, these differences highlight SAFE-AI’s pragmatic contribution, as it delivers a “minimum necessary” governance structure that is rigorous enough to support fairness, transparency, and monitoring, yet lean enough for SMEs to adopt without specialized compliance staff.

## Conclusion

The SAFE-AI ethical development framework proposed in this paper offers a practical, business-aligned approach for integrating responsible AI practices into the everyday workflows of product and data science teams. By mirroring established Agile and Scrum methodologies, SAFE-AI avoids treating ethics as a parallel or external process, instead embedding ethical evaluation directly into the structure of software and AI development. This alignment ensures that ethical considerations are addressed without compromising delivery timelines or development agility—a critical factor for SMEs operating under resource constraints.

Central to SAFE-AI is the principle that AI development introduces uncertainty and risk that cannot be adequately managed through traditional software engineering approaches alone. Accordingly, SAFE-AI emphasizes the early and ongoing use of measurable ethical metrics—acceptance criteria, fairness metrics, and transparency metrics—to evaluate model behavior. These metrics serve as practical tools for managing the probabilistic nature of AI systems, enabling teams to assess whether their models meet not only performance expectations but also ethical and social obligations.

Importantly, the SAFE-AI framework extends beyond initial deployment by incorporating a monitoring and evaluation phase that supports continuous ethical oversight. It recognizes that AI systems are dynamic and must be re-evaluated whenever retraining, tuning, or environmental shifts occur. This ensures that ethical integrity is not treated as a static checkpoint but rather as an evolving requirement that must be actively maintained throughout the product lifecycle.

A key innovation introduced by SAFE-AI is the scenario-based probability analogy mapping process as an interpretability technique designed to enhance trust among nontechnical stakeholders. Translating probabilistic model behavior into familiar, narrative-based analogies with associated risks and costs helps explain how and why AI systems reach certain predictions—especially in high-stakes health care settings where transparency is critical. Unlike traditional explainability tools that focus on model internals, SAFE-AI centers on real-world interpretability, offering an accessible way to communicate model behavior, associated risks, and uncertainty in decision-making. As such, it not only supports transparency but also serves as a bridge between ethical modeling and user-centered design.

Taken together, the contributions of SAFE-AI establish a scalable pathway for responsible AI adoption in health care and beyond where modeling excellence, software best practices, and ethical integrity are not at odds but rather deeply interwoven into a cohesive and actionable development process.

## Supplementary material

10.2196/80028 Checklist 1 SAFE-AI (Scalable Agile Framework for Execution in AI) phase summary and implementation checklist.

## Abbreviations

AI: artificial intelligence
ALE: accumulated local effects
BSA: Business Software Alliance
CCPA: California Consumer Privacy Act
DSA: Data Science Alliance
EU: European Union
FDA: US Food and Drug Administration
GDPR: General Data Protection Regulation
GenAI: generative artificial intelligence
HHS OCR: Department of Health and Human Services - Office for Civil Rights
HIPAA: Health Insurance Portability and Accountability Act
IE: inference engine
IRB: Institutional Review Board
LIME: Local Interpretable Model-Agnostic Explanations
ML: machine learning
MTN: Medical Timeseries Networks
NIST: National Institute of Standards and Technology
PDP: partial dependence plots
PE: product engine
PSI: Population Stability Index
SAFE-AI: Scalable Agile Framework for Execution in AI
SaMD: software as a medical device
SDLC: software development life cycle
SHAP: Shapley Additive Explanations
SME: small and medium-sized enterprise
SPAMM: Scenario-based Probability Analogy Mapping Method
TPLC: Total Product Life Cycle
